# Recommendations for successful involvement of patient partners in complex intervention research: a collaborative learning process

**DOI:** 10.1186/s40900-023-00533-3

**Published:** 2024-01-03

**Authors:** Pernille Christiansen Skovlund, Jeanette Finderup, Sanne Aabo, Flemming Jensen, Henning Søndergaard, Lotte Ørneborg Rodkjær

**Affiliations:** 1 Department of Oncology, Aarhus University Hospital, Palle Juul-Jensens Boulevard 99, 8200 Aarhus N, Denmark; 2 Research Centre for Patient Involvement, Aarhus University and Central Denmark Region, Aarhus, Denmark; 3 Department of Renal Medicine, Aarhus University Hospital, Aarhus, Denmark; 4 Department of Clinical Medicine, Aarhus University, Aarhus, Denmark; 5 Department of Public Health, Aarhus University, Aarhus, Denmark; 6 Department of Infectious Diseases, Aarhus University Hospital, Aarhus, Denmark

**Keywords:** Patient partners, Challenges, Initiatives, Workshops, Collaborative learning process, Recommendations

## Abstract

**Background:**

Patient and public involvement in health-related research is a new discipline in Denmark. In 2021, a national conference titled 'Patient and Public Involvement in Complex Intervention Research' provided a forum for discussion between patient partners, researchers and clinicians on involving patients as partners in complex intervention research.

**Methods:**

We aimed to describe specific challenges to and initiatives for patient partner involvement in order to develop recommendations for creating successful partnerships in complex intervention research. Through a collaborative learning process, 140 researchers identified the most important challenges for them in patient partner involvement and potential initiatives to improve such involvement. At a subsequent workshop, four patient partners identified the challenges and initiatives from their perspective as patient partners. They also gave feedback on the challenges and initiatives suggested by the researchers and helped shape three recommendations for practice. Three of the patient partners were involved in writing this paper.

**Results:**

The five most important challenges identified by researchers were time, recruitment, ethics, power and inequality. Between four and seven initiatives to overcome these challenges were suggested. The three most important challenges identified by patient partners were communication, when you get information that is hard to handle and recruitment. They suggested three to four initiatives for improvement. Patient partners confirmed the importance of all the researcher identified challenges when presented with them, they also provided additional comments on the researchers’ initiatives. This led to the formation of recommendations for involving patient partners.

**Conclusions:**

A collaborative learning process was shown to be a suitable method for patient partner involvement. Consistency was seen between the challenges and initiatives identified by researchers and patient partners. Based on these observations, three recommendations were developed: (1) create specific programmes that aim to involve all kind of patients (including but limited to vulnerable patients) as patient partners, (2) produce ethical guidelines for the involvement of patient partners, and (3) develop a national strategy for patient partner involvement. To build on these recommendations, a joint workshop with both researchers and patient partners is needed.

**Supplementary Information:**

The online version contains supplementary material available at 10.1186/s40900-023-00533-3.

## Abstract (plain language summary)

Involving patients in complex intervention research is new in Denmark, so there is a need to work out how to do it properly. In 2021, a national conference about this was arranged. There, two workshops were held with 70 complex intervention researchers in each. In the workshops, challenges and steps needed to bring patient partners into complex intervention research were identified. Later, a similar workshop was organized with four patient partners. Their views were similar to what was concluded at the earlier workshops. All challenges and steps to overcome these were discussed between patients and researchers at the last workshop. This resulted in the development of three recommendations to successfully involve patient partners into complex intervention research: (1) create programmes to involve patients who might otherwise be missed as patient partners, (2) produce ethical guidelines for involving patient partners in complex intervention research, and (3) develop a national plan for involving in patient partners.

**Supplementary Information:**

The online version contains supplementary material available at 10.1186/s40900-023-00533-3.

## Background

There is a growing interest in involving patients in health service research, and this is an increasingly accepted component of research in many countries and recognized as an important part of the research process [1]. Complex interventions [2] are often used in health services. A complex intervention refers to a multifaceted approach or set of actions designed to address complex problems or conditions, often involving multiple interacting components or factors [2]. Involving patients (e.g., stakeholders) in the development of these interventions is one of the core elements [2] of this process. Patient involvement is needed to increase the potential of developing an intervention that is likely to have positive impacts on health and to enhance the possibility of achieving changes in practice. It is important to consider how such a partnership should be established, and the appropriate methods necessary to identify and involve such stakeholders [2]. Furthermore, it has become increasingly more common for the fund committees in Denmark to look at how you have involved members of the public in developing your proposal and how you intend to involve them in your research study when applying for a research grant e.g. the Heart Association [3] and the Diabetes Association [4]. This is challenging, and researchers and stakeholders need guidance on how to build such a partnership. Generally, patient and public involvement in health-related research (PPI) refers to research carried out "with" or "by" members of the public (partners), rather than "to", "about" or "for" them (participants) [5]. PPI can be achieved in different ways, e.g., a research team can collaborate with patient partners across some or all stages of a study, from problem identification to research dissemination. PPI can also be part of the structures and institutions of research, e.g., setting research priorities [5]. On the continuum of patient involvement practices, patient partnerships involve collaboration, shared leadership practices and patient partners on research teams acting as co-investigators [5–7]. Patient partners can be involved in all stages of the research process, including writing grant applications and carrying out research activities [7]. Globally, many organizations interested in PPI have developed guidance to support researchers, such as the National Institute for Health and Care Research (NIHR) in the United Kingdom [8], Strategy for Patient-Oriented Research (SPOR) in Canada [9], Patient-Centered Outcomes Research Institute (PCORI) in the United States [10], European Patients’ Forum—*The Value* + *Handbook* [11] and International Collaboration for Participatory Health Research (ICPHR) [12]. They are all initiatives that have been developed to guide researchers in their work with PPI but under different definitions of PPI and diverse missions and visions. Furthermore, studies offer a substantial evidence base on certain aspects of the impact of PPI in health research. These studies have identified e.g., how PPI can increase recruitment to clinical trials [6, 7], make research more relevant and appropriate for users, help to formulate research questions and develop study design, and provide insights to inform the conduct of analysis [13–15].

Although there are many guidelines on how PPI should be carried out, and at least 65 frameworks have been developed for assessing the nature of and evaluating patient partnership processes, outcomes and impacts in health research [16], there are still critical barriers for researchers seeking to involve patients as partners [17, 18]. A scoping review by Bird et al. [19] highlights several barriers and facilitators for researchers to understand when using patient partnerships in the research process. Many researchers continue to struggle with how to operationalise research partnerships with patients practically and effectively. Therefore, it is critical to identify strategies that enable the optimal involvement of patients, and additional evidence is needed to understand both the researchers' and patient partners' perspectives on the patient partnership process.

In Denmark, involving patients as partners in research is still a relatively new discipline, but there is increasing focus on it. Although more and more researchers are involving patient partners in the research process, there is still a lack of knowledge about the challenges—from both the researcher and the patient partner perspectives in a Danish context—of successfully establishing this partnership. A Danish study by Skovlund et al. [20] found that patient partners with cancer contributed a new vocabulary and perspective to the dialogue, and they validated the results of the project. PPI brought to light considerations related to emotional aspects (e.g., sadness/sorrow and existential thoughts), administrative aspects (e.g., arranging meetings, balancing work and small talk) and intellectual aspects (e.g., avoiding information that harms, continuing activities despite the death of patients). Another Danish study by Finderup et al. [21]found that important facilitators of patient involvement in chronic kidney disease research included working as a team, being a part of the process and being prepared for the work. Important barriers included patient vulnerability and uremic symptoms, both of which must be considered. Even though the barriers, considerations and facilitators related to patient involvement identified in the two Danish studies were found in collaboration between researchers and patient partners, a deeper understanding of the challenges to and initiatives for improvement may be achieved through a collaborative learning process.

## Methods

This study aims to describe specific challenges to and initiatives for patient partner involvement in order to develop recommendations for creating successful partnerships [22] in complex intervention research. The study involved a collaborative learning process in which we engaged researchers and patient partners through workshops.

### PPI in the research process

Three patient partners from one of the workshops were engaged in the dissemination phase of the study with the aim of writing this paper. These patient partners provided feedback on the results and discussion sections and have been involved in developing recommendations for future practice. We have met both online and in person, being as flexible as possible, but we have not all been able to meet at the same time due to lack of time and patient partner illness. Having engaged patient partners in writing this paper, we have chosen to report it in accordance with the Guidance for Reporting Involvement of Patients and Public (GRIPP2) Short Form reporting guideline [23]. The GRIPP2 checklist was completed in collaboration with the patient partners and can be found in Additional file 1: Appendix I.

### Collaborative learning

“Collaborative learning” is an umbrella term for a variety of approaches, including a collaborative intellectual effort that participants and researchers make together. Usually, participants work in groups to create a product [22]. Collaborative learning represents a shift from a researcher-centred approach to a participant-centred approach. The outcomes of this type of learning process depend less on lectures and more on discussions and active work by the participants. In this paper, we present data collected from such discussions and active work with our participants.

### Setting

In November 2021, a national conference titled ‘Patient and Public Involvement in Complex Intervention Research’ was held in Denmark for patient partners, researchers and practitioners on engaging patient partners in complex intervention research [24]. At this conference, two identical workshops (workshops Ia & Ib) were held. Details of the workshops are given in Table 1, and the content and structure of the workshops are presented in Table 2.

**Table 1:** Details of workshops Ia & Ib and II

Workshops Ia & Ib	Workshop II
Held at a conference in November 2021	Held as a stand-alone event in April 2022
Participants: Researchers, clinicians and patients, all with research competences Learning outcomes: To gain knowledge on how to engage patient partners in complex intervention research To understand the possibilities and challenges when engaging patient partners in complex intervention research To reflect on own practice and identify areas for improvement Methods: Collaborative learning process [22]	Participants: Patient partners without academic competence Learning outcomes: To understand the possibilities and challenges when engaging patient partners in complex intervention research To reflect on patient partners’ participation in complex intervention research and compare their perspectives with those of the participants at workshops Ia & Ib To identify areas for improvement in engaging patient partners in complex intervention research Methods: Collaborative learning process [22]

**Table 2:** Content and structure of workshops Ia & Ib

Content	Format
Welcome and a brief introduction to PPI using the PPI guidance developed by the PPI research group in ResCenPI [26]	10-min lecture
Case study [21]—how to engage patient partners in all phases of the research process and the impact on patient partners	15-min presentation
Case study [20]—the challenges in the relationship between researcher and patient partner and the impact on patient partners	15-min presentation
Based on own experiences, the participants identified their challenges	5 min using an interactive presentation tool Mentimeter.com [27] all participants could write down their challenges, and everybody’s responses were shown as a word cloud, with the word mentioned most in the centre and biggest
One of the four most-mentioned challenges was picked by each group, and they collaboratively suggested initiatives to overcome their chosen challenge	15 min of group work Using a pictorial template on worksheets (shown in Fig. 1), the participants worked in groups of 6–8
The groups presented their initiatives	15 min of plenum discussion in total

Although participants at the conference comprised patient partners, researchers and practitioners, we will refer to all categories as researchers in the text that follows. The patient partners at the conference had a double competence, including an academic competence, and the case was the same for the practitioners who were also involved in doing research. After the conference, patient partners from different complex intervention research projects but without this double competence were invited to a separate workshop (workshop II). This was held in April 2022. Details of workshop II are also shown in Table 1, and the content and structure of the workshop are given in Table 3. The duration of this patient partner workshop was two hours including a break. This workshop took the form of a hybrid meeting, with options to participate either face-to-face or online via Zoom [25].

**Table 3:** Content and structure of workshop II

Content	Format
Brief introductions	All participants introduced themselves by talking about their experiences with PPI
Based on own experiences, the participants identified their challenges	Using yellow notes, the participants had 5 min to write down challenges. Afterwards, the patient partners presented their yellow notes individually and then put them in the middle of the table. Notes that mentioned the same challenge were put together
The patient partners chose three of the challenges they had mentioned and collaboratively found initiatives to overcome their chosen challenges	Using a pictorial template (shown in Fig. 1), the participants worked as a group
The patient partners validated the worksheets developed in the first two workshops on both challenges and initiatives	The worksheets developed by the participants in the two earlier workshops were shown to the patient partners. The patient partners’ reflections were added to these worksheets

### Participants

The participants in workshops Ia & Ib were affiliated with a broad range of academic (university and professional training) bodies and health organizations (e.g., hospitals, and community, patient and research establishments) from across Denmark, and all five regions of the country were represented. There was also a mix of specialisms (e.g., diabetes, rehabilitation, kidney disease, cancer, heart disease) and professions (e.g., health service researchers, educators, doctors, nurses, physiotherapists, occupational therapists). In total, 140 delegates participated in the conference. The participants in workshop II were recruited through researchers in a PPI research group embedded in the Danish Research Centre for Patient Involvement (ResCenPI) [24]. These researchers sent out an invitation to their patient partner networks. Eight patient partners replied to the invitation, and four agreed to participate. The rest declined due to lack of interest or time or because the planned schedule did not suit them. All four patient partners had prior experience as patient partners in complex intervention research, ranging from one year to 10 years. Three of the patient partners identified as males and one as female.

### Data collection

**Fig. 1:**
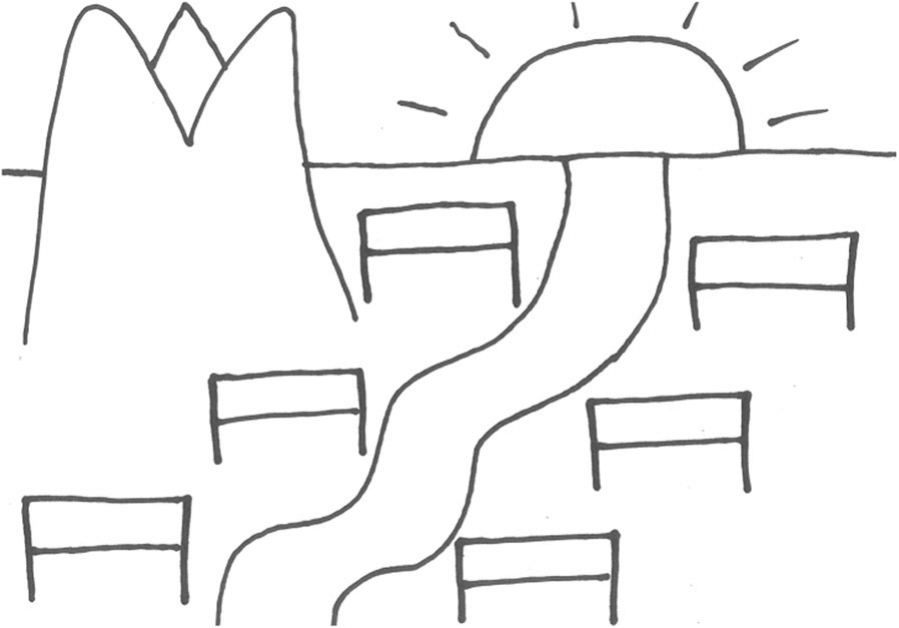
Worksheet for group work. Participants wrote their chosen challenge at the bottom of the road. To reach the sunrise—representing the point where the challenge had been overcome—initiatives had to be taken, and participants were encouraged to write their suggested initiatives on the signs along the road. They could also, as a reflection exercise, write down some of the barriers to overcome in the mountains on the left of the worksheet

The challenges reported by the participants in workshops Ia & Ib were collected using Mentimeter.com [27]. This ensured a collaborative learning process, because all the challenges chosen were shown to the participants on a screen in the form of a word cloud, with the challenge mentioned most often in the centre of the cloud. The challenges experienced by the participants in workshop II were collected using yellow notes because this workshop’s hybrid format made the use of supporting technology more difficult. The participants presented their yellow notes individually, and these notes were put in the middle of the table. As part of the collaborative learning process, some notes were put together if all participants agreed that they covered the same challenge. Workshops Ia & Ib had both such a large number of participants that the three workshop facilitators were not able to have close contact with each group. A pictorial template on a worksheet (Fig. 1) was therefore developed to encourage the participants to complete the given task in a very short time frame [28]. The pictorial template (Fig. 1) worked as a co-facilitator and did not provide an outcome as such, but was designed to ensure that each group delivered an output that could be used in the subsequent stages of the process. Although workshop II only had four participants, we chose, for reasons of consistency, to use the same pictorial template (Fig. 1) as a co-facilitator in this workshop. The completed worksheets from all the groups in workshops Ia & Ib and from workshop II were collected at the end of each session.

### Data analysis

Data from the two word clouds were imported into NVivo [29] and similar words like “ethics” and “ethical” were merged. Data from the yellow notes were merged if they covered the same challenges but had been named in different ways. Completed worksheets from workshops Ia & Ib that covered the same challenges were condensed into one worksheet for each challenge; at workshop II, these worksheets were validated by the participants, and some fresh ones were also completed.

### Ethical considerations

When running the workshops Ia & Ib, we were not aware that data from the workshops could turn into research data. Therefore, the participants in workshops Ia & Ib did not provide informed consent to participate in this study. However, the collaborative process in these workshops, the data collected by Mentimeter.com [27] and the worksheets completed in groups do not reflect the views of any identifiable individual. The participants (patient partners) in workshop II were informed that the output of the workshop would be used in a research paper. They all gave oral informed consent.

## Results

### Challenges to and initiatives for improvement in engaging patient partners in complex intervention research: the researchers' perspective

**Fig. 2:**
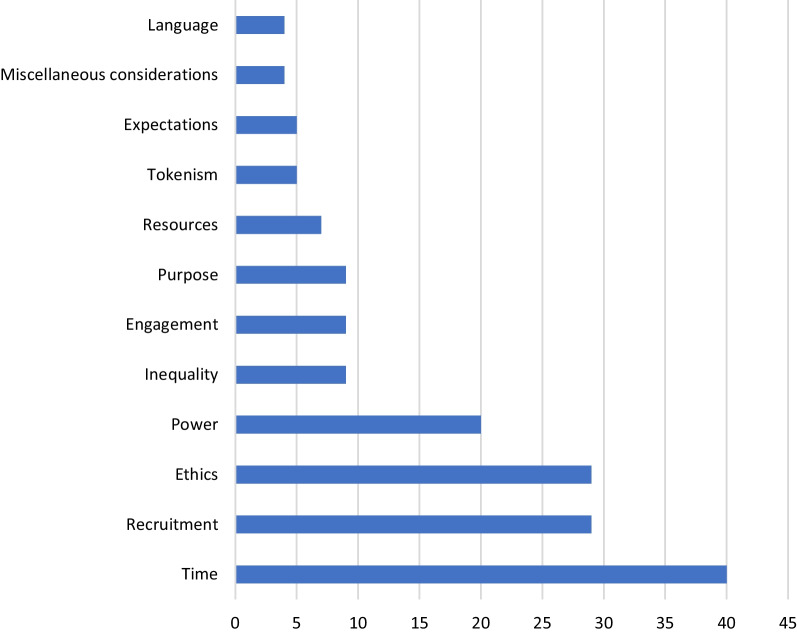
Challenges mentioned by more than two participants in workshops Ia & Ib (From a total of 309 votes, each participant could vote several times)

At workshops Ia & Ib, Mentimeter.com highlighted several challenges identified by researchers in engaging patient partners in complex intervention research (Fig. 2). The five most-chosen challenges in the two workshops were: time, recruitment, ethics, power and inequality. Table 4 shows the challenges identified, with associated initiatives for improvement. As an example, the challenge "time" is associated with the following researcher-identified initiatives: "clarification of roles and tasks", "training of both researcher and patient partner", "common focus", "structure and detailed plan", "framework and strategy" (Table 4).

**Table 4:** Challenges to engaging patient partners in complex intervention research and initiatives to address them—identified by researchers

Challenges	Time	Recruitment	Ethical issues	Power	Inequality
Initiatives towards improvement	Clarification of roles and tasks	Identification of the target group	Relevance to both the patient and the researcher, and creating value	Attention to the dialogue	Establish relationships/people skills
	Training of both researcher and patient partner	Creating a relationship	Thorough preparation and follow-up	Preparation of patient partners and researchers for the task and collaboration	Interpretation agreed by patients and researchers
	Common focus	Professional patient partners and a user panel	Code of ethics must be drawn up	Make it clear that the researcher is dependent on the participants/patient partners	Adaptation in relation to structure, time, method and flexibility
	Structure and detailed plan	Focus on patient partners' contribution in relation to recruitment	Collaboration agreement between patient and researcher	Facilitate the process so that everyone has an opportunity to participate	Contact person
	Framework and strategy	Reconciliation of expectations in relation to purpose and scope	Preparation of a confidentiality agreement	Environmental change	
		Clarification of the task		Number of participants must be considered	
		Taking the plunge even as an unexperienced researcher			

### Challenges to and initiatives for improvement in engaging patient partners in complex intervention research: the patient partners' perspective

At workshop II, 16 challenges to engaging patient partners in complex intervention research were identified from the patient partners' perspective and written on yellow notes (Table 5). Based on consensus between the four patient partners, three challenges were identified as the most important for the patient partners to discuss. These were "communication", "when you get information that is hard to handle" and "recruitment". Each of these challenges was recognizable to all four patient partners. The patient partners discussed and agreed on initiatives for improvement for their identified challenges (Table 5). As an example, the identified challenge "communication" is associated with the following patient partner-identified initiatives: "Find each other and share a common language", "communicate concisely and precisely both orally and in writing—communication is also what is not said" and "create possibilities to communicate in different ways".

**Table 5:** Challenges to engaging patient partners in complex intervention research and initiatives to address them—identified by patient partners

Challenges identified (on yellow notes)	Consensus on most important challenges	Initiatives for improvement
Roles (treated as object vs. subject) Inequality (being a partner, but we are all different) No matching of expectations regarding work burden When engagement happens too late in the process (e.g., after the research question has been defined) Recruitment (which patients) Hard to reach the project team Inflexible meeting arrangements (when and where) No contact person No communication about the process When you get information that is hard to handle Long days of working/meetings When you are not listened to	Communication	Find each other and share a common language
		Communicate concisely and precisely both orally and in writing—communication is also what is not said
		Create possibilities to communicate in different ways
	When you get information that is hard to handle	What I (patient partner) want to know and what I don’t want to know need to be outlined in a contract between patient partner and researcher
		Get help when something is difficult because everything cannot be scheduled
		Information about participating as a patient partner must make it clear that you might receive information that is difficult to handle; this may cause some to refuse to participate
		Allow the patient partner to retire temporarily during the project. Provide the possibility of an “emergency brake” where you can talk to someone about the difficult stuff you have encountered
	Recruitment	Recruit patient partners through general practitioners in order to reach a broader group of patients and not just those already in contact with the hospital
		Specify the tasks in relation to time, interest and knowledge needed to fulfil the role of patient partner
		Researcher and patient partner mutually define clear criteria about what experiences, knowledge and understanding are needed; where you are in your disease trajectory

### Validation by the patient partners of the researcher-identified challenges to and initiatives for improvement

The patient partners were shown the completed worksheets from workshops Ia & Ib. They recognized and confirmed the importance of all the researcher-identified challenges, and they were surprised that they themselves had not identified the challenge “inequality”, because it resonated with all of them. The patient partners acknowledged the researcher-identified challenge “power”, but they wanted to term it differently: “The challenge is more about securing equality than about power,” all the patient partners agreed. The patient partners also confirmed the researcher-identified initiatives for improvement. However, they expanded on these initiatives with additional comments. These are listed in Table 6. As an example of additional initiatives associated with the challenge “ethical issues”, they argued for “control/oversight of the researchers—to ensure they are using the patient partner input actively and appropriately”.

**Table 6:** Patient partners’ additional comments on the researcher-identified initiatives for improvement

Challenges	Power	Ethical issues	Social inequality	Time	Recruitment
Patients’ additional comments	The challenge is more about securing equality than about power	Control/oversight of the researchers—to ensure they are using the patient partner input actively and appropriately	It is difficult to ensure equality	Flexibility in relation to meeting times, e.g., once a month, possibly during working hours	In relation to recruitment, dropouts must also be considered
	Power over weak patients	Gender variation must be ensured	Accept that some cannot join/be engaged	The first meeting must include a clarification of expectations	Wider recruitment—e.g., general practitioners, and from patient associations and the media
	Meet somewhere else—at hotel or go out for dinner	The duty of confidentiality was emphasized	Other patients may support weak patients		Take the plunge even as an inexperienced researcher or patient partner; otherwise, you will create a block for yourself
	Preparation of the patient partners was emphasized		Translate what is being said—also afterwards		Mentor for the researcher and the patient partner
			Bringing in a contact person—this could be a clinician or another assistant with whom you can have a relationship. It can be a support person. If this is a doctor/researcher, it may block input		
			Flexibility was emphasized		

### Outcome of PPI in this study

The patient partners in this study contributed as co-authors to the analysis and interpretation of data by discussing how to present and expound data with the authors who were researchers. The patient partners revised the first manuscript of the paper critically for important intellectual content—for example, how to frame the recommendations and make the focus of these precisely. They created the first draft of the Plain English Summary after the first manuscript of the paper was discussed. All authors agreed to be accountable for all aspects of the work in ensuring that questions related to the accuracy or integrity of any part of the work are appropriately investigated and resolved. All patient partners who were co-authors contributed with a lot of energy to the writing process, but due to illness, we were not able to meet all at the same time and therefore some tasks like the Plain English Summary were produced by e-mailing back and forth.

## Discussion

Complex intervention research involves a complex partnership, which is why a collaborative approach is required to gain knowledge about how to facilitate such research. Bell et al. [30] support this approach and suggest that co-developing a strategy with all stakeholders (e.g., patients, researchers, healthcare professionals, health system decision makers) is essential as it increases the quality, realisation and reliability of the research. This study revealed both challenges to and initiatives for improvement in engaging patient partners in complex intervention research from both the researchers’ and patient partners’ perspectives in a collaborative learning process. We found several areas common to the two groups.

Challenges reported by both groups are also found in other studies. For example, the challenge “power” was also found in the scoping review by Bird et al. [19]. In that review, some of the most common barriers to patient involvement were the use of jargon, power imbalances between the patient partner and researcher, and the impact of time pressures on the research process. Other barriers were logistical hurdles and challenges with retention of partners in studies as they experienced changes in their life or disease. In addition, some of the studies reviewed reported that partnerships were found to be a burden to both patients and researchers in terms of their emotional impacts and due to a lack of financial resources [19]. Facilitators that were highlighted included the following: a clear role description, clarified responsibilities, meeting the personal needs of patient partners through scheduling adjustments, transportation and compensation for their time and work, and flexibility and responsiveness on the part of the research team [19]. These facilitators were also identified as initiatives towards improvement in our study, where flexibility in relation to meeting times and clarification of expectations were suggested by the patient partners (see Table 6). Other studies highlight similar challenges to those found in our study, and those studies underline that all of these challenges might have a negative impact on PPI and therefore on establishing successful partnerships. More attention needs to be paid to mitigating the possible negative effects of involvement for patient partners [31, 32].

Several studies highlight that power dynamics might influence the effectiveness and success of the partnership, and these remain a problematic barrier [33, 34]. In terms of “power”, Greenhalgh et al. [16] argue that we still do not know very much about whether or how PPI changes power relations between the researchers and the patient partners, because this is rarely the focus in terms of the impact of research. Greenhalgh et al. further argue that we need to address the negative impacts and the metrics that measure these, and ways to decrease inequalities, and suggest that we also need to question whether the language of measurement and impact is supportive or not in facilitating the improvement of PPI [16].

The challenge of “inequality”, mentioned by the researchers and recognized by the patient partners in our study, underlines the issue around barriers to diversity within PPI. A study by Reynolds et al. [35] argues that this challenge has consequences in terms of the patients participating in PPI, with people from lower socioeconomic groups and ethnic minorities, and those with low health literacy often excluded. This highlights the importance of more flexibility and responsiveness to the needs of people from different backgrounds and with different resources to enable them to involve in PPI.

The challenge of “ethical issues” identified in our study was mentioned as a challenge in terms of doing research with, rather than on, the patient, which would involve changes in the way we reflect on the ethics of our approach. A scoping review by Martineau et al. [36] highlights that we should broaden the ethical discussion on PPI, not only relying on a research ethics framework, but also framing it within the areas of research integrity, organizational ethics and relational ethics.

Both researchers and patient partners mentioned a need for training in PPI. The scoping review by Bird et al. [19] highlighted that one of the most common barriers across studies was the lack of training for patient partners, which underlines the need to focus on preparing both the researcher and the patient partner in how to establish the partnership and clarify their roles and expectations.

If the challenges mentioned above are not taken into account, it can have a negative impact for both the researcher and the patient partner, and for that reason, it is important to keep these areas in mind throughout the collaboration.

### Collaborative learning process

We aimed to use a collaborative learning process and found that this process worked very well to engage both researchers and patient partners in describing challenges to and framing initiatives for the successful engagement of patient partners in complex intervention research. For us as authors, this publication and its recommendations for successful engagement of patient partners in complex intervention research are the outputs of the collaborative learning process. However, for these to be outputs for all the participants, we have to share the recommendations with them [22]. We established a number of learning outcomes for the workshops. After workshops Ia & Ib, a survey was sent out to all the participants, and 96% of them reported that they had benefited from attending and found the workshop good or satisfactory [24]. However, an evaluation of how each participant achieved the learning outcomes would have been more appropriate [22]. We did not conduct any evaluation of workshop II, but three patient partners agreed to continue the collaboration by co-authoring this paper. The collaborative learning process might be a method to consider when including PPI in complex intervention research. It produces a high level of engagement, as outlined in Arnstein’s ladder of participation [37] and the achievement of a personal outcome, and not just a research outcome, signifies meaningful engagement in PPI [38]. Despite this, a literature search only revealed two projects that used both PPI and a collaborative learning process [39], and both are reported in the same publication. One was about changing practice in dementia care, and the other dealt with changing practice in care for patients with septicaemia in a hospital setting. Both could be considered to involve complex interventions [2]. Our study’s main goal was to develop recommendations for PPI practice, and the goal of changing practice is what it shares with these two previous projects. Therefore, it may be the case that the collaborative learning process is especially useful in PPI if the goal is to change practice.

## Limitations

The findings in this paper reflect a Danish setting and factors affecting the implementation of PPI in Denmark. The collaborative learning process at workshops Ia & Ib was influenced by the two case studies [20, 21] that were presented at the workshops. The 140 researchers therefore had these in mind when they listed the challenges they could identify in engaging patient partners in complex intervention research. The patient partner participants had no direct discussion with the researcher participants, only with the three researchers who were authors of this paper and organized all three workshops. The patient partner participants reflected on the responses of the researcher participants, but not the other way round. The most important limitation of this study is that it was not initially designed as a research project, which is why some of the processes were not planned systematically and with PPI throughout the entire research process. Another important limitation is that the suggested recommendations for changing practice, which follow, have not yet been tested. Further on, we did not obtain informed consent from the participants in the researcher workshop, due to a lack of awareness considering these workshops as research. None of the data is indefinable, but the results reported in this paper has a potential to increase the knowledge about initiatives to overcome challenges regarding PPI, why we were obliged to report it.

## Recommendations for changing practice

The strong correspondence between the challenges found by researchers and patient partners respectively underlines the importance of finding ways to overcome these challenges in practice. The participants at the workshops provided excellent suggestions for new initiatives, which yielded three specific actions to be applied in practice:

1. *Create specific programmes that aim to involve all kinds of patients as patient partners.* Such programmes could minimize the challenge of “inequality” by supporting partnerships between researchers and patients who are not normally involved in such partnerships. In a study by Ocloo and Matthews, the authors stated that equality and discrimination barriers for involvement were on the basis of gender, ethnicity, culture, belief, sexuality, age, disability and class [33]. As Denmark has a relatively low income, gender and sexuality inequality [40], our challenge is foremost on supporting vulnerable patients in partnerships (e.g., patients who are from socioeconomically deprived areas, patients from ethnic minority communities, or patients who are otherwise marginalized or disadvantaged in our society). There is an example of such a programme in Ottawa, Canada, where First Nations, Inuit and Metis women have partnered with researchers to develop a collaborative framework defined by community members and their research partners together as ethical, useful and relevant [41]. An additional operational initiative to address inequality could be the establishment of a partnership between vulnerable patients and a contact person knowledgeable about the research project. This could be a designated person within the research project that the patient partner could turn to for advice or help in the process. Such a contact person could become a trusted individual for the patient partner. A trusted individual has also been suggested in a systematic review of 32 empirical studies about involving vulnerable groups in the co-production of research [42]. In that case, the authors proposed eight heuristic principles to overcome the challenges to the involvement of vulnerable patients. One of these principles concerns fostering well-being by encouraging vulnerable patient partners to discuss involvement with a trusted individual or even to bring them along to meetings. NGO representatives, adult gatekeepers and more experienced peers could all be potentially useful sources of practical and emotional support [42]. As suggested by both researchers and patient partners at the workshops in our study, a contact person/trusted individual could focus on fostering relationships that enable participation by vulnerable patients. This could be done by preparing patients for meetings with the project team and other patient partners, helping to interpret or translate questions from the researchers, and keeping up with meeting details and other aspects of patient partners’ involvement in a complex intervention research project. A future workshop in Denmark for both researchers, patient partners, patient organisations, life science companies and leaders from universities and university hospitals to address the establishment and content of such programmes would be highly relevant. As members of ResCenPI, taking a lead on PPI in Danish health research, we acknowledge our obligation to facilitate such a workshop, and we plan to carry this out when funding has been secured. The authors of a recent systematic review exploring the theory, barriers and enablers related to patient and public involvement across health, social care and patient safety—Ocloo et al. [43]—conclude that addressing equality and diversity in relation to PPI is a neglected area. We want to change that in Denmark.
2. *Produce ethical guidelines for the Involvement of patient partners.* Our results indicate a need for some kind of ethical review process dealing with questions, concerns and perhaps even formal checks on PPI in health research. In Denmark, there is no requirement for researchers to seek ethical approval for public involvement in research [44]. Nevertheless, there is a need for a forum to debate all the ethical issues surrounding PPI. Challenges to be discussed could include how researchers work with patients and the public throughout a research project in an ethically appropriate way, and how researchers take care of patients if they receive information that is hard to handle. Research communities like ResCenPI could produce a code of ethics or develop ethically conscious standards, including terms of confidentiality, if the regulatory authorities do not make such a service available. Pandya-Wood et al. have developed a framework for public involvement at the design stage of NHS health and social care research in the UK [45]. This framework was developed to help researchers recognize the ethical issues and find ethically conscious approaches to engaging the public. Such a framework is needed in Denmark, and we believe that patient partners could contribute to its development. However, action from regulatory authorities such as the scientific ethics committee is needed to create a change in the approval procedure on studies involving patients as partners in health research.
3. *Develop a national strategy for patient partner involvement.* We suggest a future project should be conducted that focuses on creating a national strategy for PPI in Denmark, with the purpose of creating a common, standardised and generic set of materials that will align the understanding of PPI across different organizations. For such a study to have an impact, the Danish Board of Health and other stakeholders from patient organisations and life science companies should be involved. In 2011, the Canadian Institutes of Health Research (CHIR) developed such a strategy, called SPOR, to empower patients within their roles in health research and the healthcare system. They intended the related materials to be generic so that they could be aligned with any individual context [9]. This strategy could serve as a starting point for other countries' respective PPI strategies—including Denmark’s.

All three recommendations underpin the importance of a multi-stakeholder approach in moving forward with PPI in Denmark. Communicating these recommendations and concerns to involved stakeholders, research funders, industry participants, academia, patient organizations and government agencies could perhaps help pave the way for greater collaboration about PPI in Denmark. Such a multi-stakeholder interaction is complex but may be able to produce synergy due to the heterogeneity that reflects critical dimensions of the barriers in PPI in Denmark from different point of views. The multi-stakeholder interaction can allow for timely adaptations to interventions [46]. The initiative to establish and drive these multi-stakeholder partnerships comes mostly from research teams [47], which indicate that moving forward, ResCenPI play a large role in reaching out and establishing such partnerships.

## Conclusion

In two workshops (Ia & Ib), 140 researchers identified the following challenges to engaging patient partners in complex intervention research as the most important: time, recruitment, ethical issues, power and social inequality. At a subsequent workshop, four patient partners identified the following as three important challenges: communication, when you get information that is hard to handle and recruitment. The patient partners reviewed the researcher-identified challenges, and these all resonated with them. At all three workshops, initiatives for ensuring successful patient partner involvement in complex intervention research were identified. There was consistency in the challenges and initiatives identified by both researchers and patient partners. Based on these challenges and initiatives, three recommendations were developed between the patient partners and the organizers of the workshops: (1) create specific programmes that aim to involve all kinds of patients (including vulnerable patients) as patient partners, (2) produce ethical guidelines for the involvement of patient partners, and (3) develop a national strategy for patient partner involvement. The focus of these were stated clearly and precisely by discussing them within the author group consisting of patient partners and researchers. To develop these recommendations further, a joint workshop with both researchers and patient partners is needed. The collaborative learning process was a consistently useful method for facilitating all three workshops. This method was shown to be suitable for use in patient partner involvement.

### Supplementary Information

**Additional file 1:** The GRIPP2 checklist was completed in collaboration with the patient. partners.

## Data Availability

Data are available upon reasonable request.

## Abbreviations

CHIR: Canadian Institutes of Health Research
GRIPP2: Guidance for Reporting Involvement of Patients and the Public
ICPHR: International Collaboration for Participatory Health Research
NIHR: National Institute for Health and Care Research
PPI: Patient and public involvement in health-related research
PCORI: Patient-Centered Outcomes Research Institute
ResCenPI: Research Centre for Patient Involvement
SPOR: Strategy for patient-oriented research
