# Supplementary material for: Recommendations for successful involvement of patient partners in complex intervention research: a collaborative learning process
Source: Res Involv Engagem. 2024 Jan 3;10:3. doi: 10.1186/s40900-023-00533-3 (PMC10765637; doi:10.1186/s40900-023-00533-3)
Supplement: Supplementary file 1 — Additional file 1: The GRIPP2 checklist was completed in collaboration with the patient. partners. [file 40900_2023_533_MOESM1_ESM.docx]

###### GRIPP2 short form

Staniszewska S, Brett J, Simera I, Seers K, Mockford C, Goodlad S, Altman DG, Moher D, Barber R, Denegri S, Entwistle A, Littlejohns P, Morris C, Suleman R, Thomas V, Tysall C. GRIPP2 reporting checklists: tools to improve reporting of patient and public involvement in research. BMJ. 2017 Aug 2;358:j3453.

| Section and topic | Item | Reported on page No |
| --- | --- | --- |
| 1: Aim | Report the aim of PPI in the study | 4 |
| 2: Methods | Provide a clear description of the methods used for PPI in the study | 4 |
| 3: Study results | Outcomes—Report the results of PPI in the study, including both positive and negative outcomes | 12 |
| 4: Discussion and conclusions | Outcomes—Comment on the extent to which PPI influenced the study overall. Describe positive and negative effects | 4, 17 |
| 5: Reflections/critical perspective | Comment critically on the study, reflecting on the things that went well and those that did not, so others can learn from this experience | 12 |

PPI=patient and public involvement
